# Supplementary material for: The genome sequence of Prunus padus L., 1753 (Rosales: Rosaceae)
Source: Wellcome Open Res. 2026 Jun 2;11:333. [Version 1] doi: 10.12688/wellcomeopenres.26679.1 (PMC13563005; doi:10.12688/wellcomeopenres.26679.1)
Supplement: Peer review history

# Reviewer report: Reviewer response for version 1

**DOI:** 10.21956/wellcomeopenres.29380.r164534

**Published:** 2026-08-31

**Authors:** Sunil Kumar Sahu

**Affiliations:** 1 State Key Laboratory of Genome and Multi-omics Technologies, BGI Research, Shenzhen, China

This Data Note presents a valuable, openly accessible chromosome-level genome assembly of *Prunus padus.* The dataset is potentially useful for comparative genomics, polyploidy research, genome evolution, trait-associated studies, and future annotation work. The sequencing strategy, assembly workflow, curation procedures, quality assessment, and data-access information are generally appropriate and technically sound. The primary assembly spans 454.49 Mb, with 97.36% assigned to 16 chromosomal pseudomolecules, a scaffold N50 of 27.2 Mb, and a consensus QV of approximately 60.8. However, several issues should be addressed before the Data Note is considered fully satisfactory.

 1. The primary assembly contains 99.1% complete BUSCOs, but only 16.5% are reported as single-copy and 82.6% as duplicated. This strongly deviates from the benchmark shown in Table 4, where duplicated BUSCOs are expected to be below 5%. The manuscript reports this result but does not adequately explain it. Given the reported chromosome number of 2n=322n = 322n=32, the presumed base chromosome number of x=8x = 8x=8, and the likely tetraploid nature of *P. padus*, elevated duplication may reflect genuine genome duplication, retained homeologous regions, or incomplete haplotype separation. The current wording may lead readers to interpret the high duplication rate as a quality failure, whereas it could be biologically meaningful. This distinction should be made explicit.

 2. The manuscript states that library preparation was modified so that it occurred while DNA remained bound to enrichment beads. However, the specific modifications to the NEBNext Ultra II workflow are not described, and no protocol or application note is cited for this procedure.

 3. The Methods section inconsistently reports suppliers, manufacturers, and geographic locations. Some reagents and instruments include the manufacturer but not the country, whereas other sections provide more complete information. Please apply a consistent format throughout. 

 4. Two mitochondrial sequences of 280.28 kb and 155.92 kb are reported. The authors should briefly explain whether these represent alternative mitochondrial configurations, separate circular molecules, branched graph-derived sequences, or distinct contigs retained because of structural ambiguity.

Are sufficient details of methods and materials provided to allow replication by others?

No

Is the rationale for creating the dataset(s) clearly described?

Yes

Are the datasets clearly presented in a useable and accessible format?

Yes

Are the protocols appropriate and is the work technically sound?

Yes

Reviewer Expertise:

Plant genomics and evolution

I confirm that I have read this submission and believe that I have an appropriate level of expertise to confirm that it is of an acceptable scientific standard, however I have significant reservations, as outlined above.

# Reviewer report: Reviewer response for version 1

**DOI:** 10.21956/wellcomeopenres.29380.r164537

**Published:** 2026-08-21

**Authors:** Samuel J. Smit

**Affiliations:** 1 University of York, York, England, UK

The authors provide a high quality, chromosome level assembly of *Prunus padus*, a widely distributed member of the Rosaceae. The assembly methods and data generated are clearly described, technically sound and reproducible.

 The comments below reflect very minor issues with the manuscript and can be easily addressed.

- For consistency throughout the Methods section please include details on supplier/manufacturer and country for all referenced products, instruments and kits. Under the Nucleic acid extraction section these details are missing with subsequent sections missing the country.
- Under the Nucleic acid extraction section (Pacific Biosciences) precedes AMPure PB beads.
- BUSCO scores in Figure 5 show duplicated BUSCO genes at 82.6%, suggesting that BUSCO was performed on the combined assembly. Can the authors provide an assessment of both the primary and alternate assemblies in Table 4? This will provide a more complete summary of the haplotypes and enable an objective comparison on the quality of the alternate assemblies.

Are sufficient details of methods and materials provided to allow replication by others?

Yes

Is the rationale for creating the dataset(s) clearly described?

Yes

Are the datasets clearly presented in a useable and accessible format?

Yes

Are the protocols appropriate and is the work technically sound?

Yes

Reviewer Expertise:

plant genome assembly, genome annotation, comparative genomics

I confirm that I have read this submission and believe that I have an appropriate level of expertise to confirm that it is of an acceptable scientific standard.

# Reviewer report: Reviewer response for version 1

**DOI:** 10.21956/wellcomeopenres.29380.r161883

**Published:** 2026-08-19

**Authors:** Andrea Menicucci

**Affiliations:** 1 University of Bologna, Bologna, Italy

**Manuscript summary**

 This Data Note reports a chromosomally complete genome assembly of *Prunus padus* (bird cherry), a widely distributed temperate/boreal Rosaceae tree, generated from a specimen collected in Edinburgh, Scotland. The assembly (454.49 Mb, 16 chromosomal pseudomolecules, plus mitochondrial and plastid genomes) was produced as part of the Darwin Tree of Life (DToL) project, which aims to sequence reference genomes for eukaryotic species found in Britain and Ireland, and supersedes a previously available contig-level assembly (GCA_024362665.1) from Seoul National University.

**Minor comments:**

- The Background section gives extensive ecological and taxonomic context for *P. padus* (distribution, pollination/dispersal biology, chemical defences, ploidy at 2n = 32) and states plainly: "We present a chromosomally complete genome sequence for *Prunus padus*, as part of the Darwin Tree of Life Project," noting that a prior contig-level assembly exists (GCA_024362665.1). This establishes that the resource fills a gap (no chromosome-level reference existed) Given the extensive discussion of cyanogenic glycosides, extrafloral nectaries, tetraploidy, and herbivore interactions in the Background, the authors have material to draw a more direct line between these traits and concrete uses of the new genome (e.g., enabling comparative genomics of polyploidy in Prunus, or genetic studies of chemical defence/host-plant interactions). I recommend adding 1–2 sentences explicitly connecting the described biology to anticipated uses of the assembly.

 

- The methodology (flow cytometry, DNA barcoding, HMW DNA extraction, PacBio HiFi + Hi-C + RNA-seq, Hifiasm assembly, YaHS scaffolding, OATK for organelles, manual curation via PretextView/HiGlass, QC via BUSCO/MerquryFK/BlobToolKit) is standard, well-established DToL pipeline and is technically sound overall. Quality metrics largely meet or exceed the Earth BioGenome Project (EBP) benchmarks summarised in Table 4: consensus QV of 60.8 (benchmark ≥40), scaffold N50 of 27.2 Mb assigned to chromosome level (97.36% of assembly, benchmark ≥90%), and contig N50 of 3.5 Mb (benchmark ≥1 Mb). However, Table 4 also shows BUSCO duplication at 82.6% against a stated benchmark of D < 5% (with only 16.5% single-copy), a large deviation that is reported in the Genome sequence report ("single = 16.5%, duplicated = 82.6%") but never discussed or interpreted in the text. Given the manuscript's own note that *P. padus *is likely tetraploid (2n = 32, base x = 8), this duplication is plausibly a genuine biological signature of polyploidy/haplotype retention in the primary assembly rather than an assembly artefact, but the authors should say so explicitly rather than leaving the reader to infer it from an unremarked benchmark failure.

 
**Revision requests:  **

- Add a sentence in the Assembly quality metrics or Genome sequence report section explaining the elevated BUSCO duplication in light of the species' tetraploid status (or an alternative explanation, e.g., retained haplotig content, if that is the true cause), and note the resulting deviation from the EBP benchmark in Table 4's discussion.

 

- The Hi-C library preparation is described as following "a modified protocol where library preparation occurs while DNA remains bound to the Enrichment beads" (Hi-C library preparation and sequencing section), but the manuscript does not specify which steps of the standard NEBNext Ultra II DNA Library Prep Kit workflow were altered to accommodate on-bead processing (e.g., omitted or reordered clean-up/incubation steps), nor does it cite a written protocol (e.g., protocols.io, or an Arima Genomics application note) describing this modification. A similar unspecified modification appears earlier in the same Hi-C section, where nuclei isolation is described as "a modified protocol based on the Qiagen QProteome Cell Compartment Kit ... in which only the Lysis and CE2 buffers were used, with QIAshredder spin columns" — here the modification is at least partially specified, unlike the library-prep step. Given that the rest of the Methods is highly detailed and even links to protocols.io for other steps (e.g., HMW DNA extraction, Howard et al., 2025), this is a specific, fixable gap: the authors should either cite a specific protocol reference for the on-bead library-prep modification or add a sentence describing exactly which standard steps were changed, so the method is fully reproducible by another lab.

 

- Minor formatting inconsistency in Methods section in manufacturer name/location citation.

Are sufficient details of methods and materials provided to allow replication by others?

Partly

Is the rationale for creating the dataset(s) clearly described?

Partly

Are the datasets clearly presented in a useable and accessible format?

Yes

Are the protocols appropriate and is the work technically sound?

Partly

Reviewer Expertise:

Comparative genomics

I confirm that I have read this submission and believe that I have an appropriate level of expertise to confirm that it is of an acceptable scientific standard, however I have significant reservations, as outlined above.
